# Utilization of sperm cryopreservation in patients with testicular cancer

**DOI:** 10.1007/s00432-024-05725-2

**Published:** 2024-04-17

**Authors:** Viktoria Menzel, Emilia Richter, Charlotte Helke, Björn Thorben Bürk, Holger H. H. Erb, Steffen Leike, Angelika Borkowetz, Christian Thomas, Martin Baunacke

**Affiliations:** Department of Urology, Medical Faculty Carl Gustav Carus, TU Dresden, Fetscherstr. 74, 01307 Dresden, Germany

**Keywords:** Testicular cancer, Sperm bank, Cryopreservation, Sexual quality of life, Infertility, Cancer survivor

## Abstract

**Purpose:**

We assessed factors that affect the utilization of sperm cryopreservation before 2021, when patients covered expenses, and the influence on quality of life.

**Methods:**

Between 2011 and 2021, testicular cancer survivors (TCS) at our clinic completed a questionnaire, including EORTC QLQ-TC26, covering sperm cryopreservation, sociodemographic details, post-treatment births, and artificial insemination.

**Results:**

After 5.7 ± 3.0 years, 279 participants (64%) responded to the questionnaire. Among them, 33% (91/279) of testicular cancer survivors chose sperm cryopreservation prior to treatment, with 11% (10/91) using it for insemination. Conversely, 2% (3/188) without cryopreservation reported unfulfilled desire to have children. Univariate analysis showed TCS with cryopreservation were younger (30.6 ± 7.1 (35 (21–59)) vs. 42.4 ± 10.9 (48 (22–81)) years; *p* = 0.001), had a lower BMI (24.2 ± 3.3 vs. 26.6 ± 4.6 kg/m^2^; *p* = 0.009) and a lower Charlson Score (> 3: 36% vs. 60%; *p* < 0.001). Multivariate analysis revealed older age (≥ 37 years: OR 13.1 (5.5–31.2), *p* < 0.001) and lower education (middle school or less: OR 3.3 (1.6–6.9), *p* = 0.001) as independent factors associated with not undergoing cryopreservation. Regarding quality of life, multivariate analysis identified a lower infertility anxiety score (OR 4.3 (2.0–9.0), *p* < 0.001) and higher age (≥ 44 years: OR 5.4 (2.6–11.3); *p* < 0.001) as predictors for the absence of prior cryopreservation.

**Conclusions:**

Age and education seem to impact the choice of undergoing paid sperm cryopreservation. Urologists should inform testicular cancer patients about costs and coverage. Importantly, the occurrence of unmet desires for parenthood is minimal among those who forego cryopreservation.

## Introduction

Testicular cancer (TC) is the most common type of cancer in men between the ages of 20–35 years. In the past years, it has been reported that the incidence of TC has significantly increased in the industrialized nations (Huyghe et al. 2004; Park et al. 2018). Around a third of the patients have metastases when primarily being diagnosed. In localized state, first therapy option is surgery. If cancer has already spread, the standard treatment is a triple chemotherapy regimen consisting of bleomycin, etoposide and cisplatin (Yamashita et al. 2021). Even though patients with TC have a very good prognosis and excellent cure rate of 70–85%, the young patients often do not have fulfilled their family planning yet at the time of diagnosis (Kuczyk et al. 2000). As sexual dysfunction and infertility are important potential side effects after chemotherapy, these issues have to be addressed before start of treatment (Arai et al. 1997).

Even though available treatment modalities have increased a lot, the paternity rates in patients with TC are still reduced by an average of 15–30% compared to the general population. Moreover, a significant impairment of semen quality has been reported in patients with TC (Petersen et al. 1999).

In past studies, the most appropriate time for cryopreservation of semen has been investigated. It was observed that sperm banking should be initiated before definitive surgical treatment because it was reported that in a significant number of patients the semen quality was further reduced after surgery compared to pre-treatment values (Kuczyk et al. 2000). Moreover, it has been investigated that fertility decreases with increasing number of chemotherapy (CT) cycles (Yamashita et al. 2021).

Up to 2021, there was no legal definition regarding the financial aspects of cryopreservation in patients with statutory health insurance in Germany. The G-BA (Gemeinsamer Bundesausschuss—Federal Joint Committee Germany) directive has been regulating the details of entitlement to benefits as well as the requirements for doctors and reproductive medicine facilities on behalf of the legislature since July 1st, 2021 (Bundesausschuss 2022). Since July 2021, cryopreservation has been funded by health insurance for women up to the age of 40 and for men up to the age of 50 prior to undergoing potentially germ cell-affecting therapy. A minimum age for use of cryopreservation is not determined.

Therefore, we aimed to analyze the utilization of cryopreservation and to compare socioeconomic parameters and quality of life of TC patients with and without cryopreservation before the legislative change. With these parameters, we want to estimate the future impact on cryopreservation as a benefit of statutory health insurance.

## Methods

Study participants were testicular cancer patients treated at the Department of Urology, Medical Faculty Carl Gustav Carus, TU Dresden, between 2011 and 2021. All testicular cancer patients were included regardless of oncologic stage or treatment modality. Regularly all patients with suspicion of TC were offered cryopreservation before the start of therapy in our center. All study participants had to bear the costs of cryopreservation themselves (freezing 342 €; storing 391 €/year). To assess both the quality of life and the preservation of sperm, we dispatched questionnaires to these individuals via postal mail in March 2022. The 36-question survey, including 26 from the EORTC Testicular Cancer questionnaire, took approximately 15–20 min to complete. Patients received oral briefings from doctoral candidates before participation. Data collection occurred 2 months post-questionnaire distribution, with additional follow-up for non-responders for up to 2 months. We solicited data encompassing sociodemographic parameters, patterns of information seeking, prior utilization of sperm cryopreservation in anticipation of TC treatment, and the subsequent use of cryopreserved sperm stemming from unfulfilled aspirations for parenthood. Patient’s records were used to obtain information on the patient’s disease and treatment such as clinical stage, prognosis group, therapy and Charlson score (a method for classifying comorbid conditions which might alter the risk of mortality prior to surgery) (Charlson et al. 1987; Quan et al. 2011).

To evaluate quality of life and sexual activity, we used validated questionnaires: EORTC (European Organization for Research and Treatment of Cancer), QLQ-TC26 (Subscales Infertility, Communication, Body image problems, Job problems, Family problems, Sexual activity, Sexual problems and Sexual enjoyment), QLQ-C30 (Subscales Global Health and Quality of life) and PHQ-4 (Patient Health Questionnaire-4), which are validated screening tools for anxiety and depression (Holzer 2023). EORTC has two interpretation scales: Functional scale (low score = poor functioning, high score = good functioning): communication, sexual activity, sexual enjoyment; Symptom scale (low score = fewer symptoms, high score = more symptoms): job problems, family problems, infertility anxiety, sexual problems.

Data were analyzed using the Chi^2^-test, *t*-test and multivariate analysis. Logistic regression models were used for the multivariate estimation of risks and to predict the outcome events. *P* ≤ 0.05 was considered to indicate significance. All calculations were performed with “IBM SPSS Statistics 28” (Armonk, New York, USA). Incomplete surveys, without a minimum question completion requirement, were included, with the lowest completion being 16 out of 36 questions, including just 6 from the EORTC questionnaire. The study was approved by the local ethics committee (BO-EK-582122021). Ethical approval involved consultation with a data protection officer, ensuring secure data handling.

## Results

### Collective

From 2011 to 2021, 485 patients with TC were treated in our clinic. After a mean follow-up of 5.7 ± 3.0 years following the decision for cryopreservation after the initial diagnosis of testicular cancer, 1% (6 patients) had died, and 9% (46 TCS) had moved to an unknown address. The return rate of the questionnaire was 64% (279 out of 433). Among respondents, 28% (78 out of 279) of TCS had a clinical stage of II or III. In addition, 39% (109 out of 279) of TCS received chemotherapy with ≥ 2 cycles, and 13% (37 out of 279) underwent a retroperitoneal lymph node dissection (refer to Table 1).

**Table 1:** Overview of the collective (*n* = 279)

	All (*n* = 279)	No cryopreservation (*n* = 188)	Cryopreservation (*n* = 91)	*p* value
Age at first diagnosis (years)	38.6 ± 11.3 37 (16–74)	42.4 ± 10.9 43 (16–74)	30.6 ± 7.1 30 (18–53)	**0.001**
Follow-up year (years)	5.7 ± 3.0 6 (1–13)	6.0 ± 3.0 6 (1–13)	5.2 ± 2.9 5 (1–11)	**0.02**
BMI (kg/m^2^)	25.8 ± 4.4 25 (17–48)	26.6 ± 4.6 26 (17–48)	24.2 ± 3.3 24 (18–35)	**0.009**
Charlson score				
2	133 (48%)	75 (40%)	58 (64%)	** < 0.001**
≥ 3	145 (52%)	113 (60%)	33 (36%)	
Histology				
Seminoma	207 (74%)	144 (77%)	63 (69%)	0.2
Nonseminoma	72 (26%)	44 (23%)	28 (31%)	
Clinical stage				
I	201 (72%)	130 (69%)	71 (78%)	0.3
II	49 (18%)	36 (19%)	13 (14%)	
III	29 (10%)	22 (12%)	7 (8%)	
Prognosis group (*n* = 77)				
Good	60 (78%)	42 (74%)	18 (90%)	0.2
Intermediate	9 (12%)	9 (16%)	0 (0%)	
Poor	8 (10%)	6 (10%)	2 (10%)	
Number of chemotherapy cycles				
No	81 (29%)	53 (28%)	28 (31%)	0.9
1	89 (32%)	61 (32%)	28 (31%)	
≥ 2	109 (39%)	74 (40%)	35 (38%)	
Retroperitoneal lymphadenectomy				
Yes	37 (13%)	167 (89%)	75 (82%)	0.1
No	242 (87%)	21 (11%)	16 (18%)	

The values marked in bold are statistically significant values

### Use of cryopreservation

Before starting therapy, 33% (91 out of 188) of TCS utilized cryopreservation. In univariate analysis, patients who used cryopreservation were younger (30.6 ± 7.1 vs. 42.4 ± 10.9 years, *p* = 0.001), had a lower BMI (24.2 ± 3.3 vs. 26.6 ± 4.6, *p* = 0.009), a lower Charlson score (≤ 2: 64% vs. 40%, *p* < 0.001), had fewer children prior to therapy (69% vs. 82%; *p* = 0.02), had a high school degree (70% vs. 25%, *p* < 0.001), and a higher income (> 4000 €/month: 43% vs. 20%, *p* < 0.001) (see Table 2). In multivariate analysis, higher age (≥ 37 years: OR 13.1 (5.5–31.2), *p* < 0.001) and a lower level of education (middle school or less: OR 3.3 (1.6–6.9); *p* = 0.001) were independent predictors for not utilizing cryopreservation (refer to Table 3).

**Table 2:** TCS by use of cryopreservation (*n* = 279)

	All (*n* = 279)	No cryopreservation (*n* = 188)	Cryopreservation (*n* = 91)	*p* value
Age at survey (years)	44.3 ± 11.9 44 (21–81)	48.4 ± 11.4 48 (22–81)	35.8 ± 7.5 35 (21–59)	** < 0.001**
School degree (*n* = 278)				
Middle school degree or less	160 (58%)	162 (75%)	27 (30%)	** < 0.001**
High school degree	118 (42%)	55 (25%)	63 (70%)	
Financial income (€/month) (*n* = 250)				
< 1500	30 (12%)	22 (14%)	8 (9%)	** < 0.001**
1500–4000	150 (60%)	108 (66%)	42 (48%)	
> 4000	70 (28%)	33 (20%)	37 (43%)	
Health insurance				
Statutory	247 (89%)	170 (90%)	77 (85%)	0.2
Private	32 (11%)	18 (10%)	14 (15%)	
Family status (*n* = 278)				
Partnership	191 (69%)	131 (70%)	60 (66%)	0.5
Single	87 (31%)	56 (30%)	31 (34%)	
Children before diagnosis				
Yes	217 (78%)	154 (82%)	63 (69%)	**0.02**
No	62 (22%)	34 (18%)	28 (31%)	
Children after therapy				
Yes	42 (15%)	10 (5%)	32 (35%)	** < 0.001**
No	237 (75%)	178 (95%)	59 (65%)	
Unfulfilled desire to have children				
Yes	12 (4%)	3 (2%)	9 (10%)	**0.001**
No	267 (96%)	185 (98%)	82 (90%)	
TC26 job problems (*n* = 268)	27.2 ± 26.1 16.7 (0–100)	26.7 ± 25.9 16.7 (0–100)	28.9 ± 26.4 33.3 (0–100)	0.7
TC26 family problems (*n* = 273)	36.3 ± 35.3 33.3 (0–100)	35.5 ± 35.3 33.3 (0–100)	37.8 ± 35.6 33.3 (0–100)	0.6
TC26 infertility anxiety (*n* = 278)	33.0 ± 37.7 33.3 (0–100)	22.8 ± 33.7 0 (0–100)	53.8 ± 37.1 33.3 (0–100)	** < 0.001**
TC26 communication (*n* = 271)	81.7 ± 23.9 83.3 (0–100)	78.9 ± 26.3 83.3 (0–100)	87.4 ± 16.9 100 (33.3–100)	**0.002**
TC26 body image problems (*n* = 278)	21.8 ± 30.2 0 (0–100)	23.4 ± 31.0 0 (0–100)	18.7 ± 28.2 0 (0–100)	0.2
TC26 sexual activity (*n* = 273)	64.7 ± 26.5 66.7 (0–100)	60.1 ± 27.2 66.7 (0–100)	73.8 ± 22.4 66.7 (16.7–100)	** < 0.001**
TC26 sexual problems (*n* = 247)	19.9 ± 27.3 0 (0–100)	23.4 ± 29.3 16.7 (0–100)	13.6 ± 22.0 0 (0–100)	**0.003**
TC26 sexual enjoyment (*n* = 238)	79.3 ± 25.3 83.3 (0–100)	75.1 ± 27.3 83.3 (0–100)	87.1 ± 19.0 100 (16.7–100)	** < 0.001**
PHQ-4 (*n* = 266)	2.1 ± 2.2 2 (0–12)	2.1 ± 2.3 2 (0–11)	2.0 ± 2.1 2 (0–12)	0.8
C30 global health (*n* = 274)	3.8 ± 1.2 4 (2–7)	4.0 ± 1.2 4 (2–7)	3.6 ± 1.1 3 (2–7)	**0.02**
C30 quality of life (*n* = 273)	3.8 ± 1.2 4 (2–7)	3.8 ± 1.2 4 (2–7)	3.6 ± 1.2 3 (2–7)	0.06

The values marked in bold are statistically significant values

**Table 3:** Multivariate analysis concerning parameters for no use of cryopreservation

Parameter	Univariate analysis		Multivariate analysis	
	OR	*p* value	OR	*p* value
Age at first diagnosis (≥ 37 years)	15.1 (7.5–30.5)	** < 0.001**	13.1 (5.5–31.2)	** < 0.001**
Median BMI (≥ 25)	2.7 (1.6–4.7)	** < 0.001**	0.5 (0.3–1.1)	0.08
Charlson score (≥ 3)	2.6 (1.6–4.4)	** < 0.001**	1.5 (0.7–3.0)	0.3
School degree (middle school or less)	5.3 (3.1–9.3)	** < 0.001**	3.3 (1.6–6.9)	**0.001**
Average income (< 4000€/month)	2.9 (1.6–5.2)	** < 0.001**	2.0 (0.9–1.1)	0.09
Children before diagnosis	1.0 (1.1–3.6)	**0.02**	1.1 (0.5–2.5)	0.8
Family status (single)	1.2 (0.7–2.1)	0.5		
Health insurance (statutory)	1.7 (0.8–3.6)	0.2		

The values marked in bold are statistically significant values

### Wish to have children after therapy

After testicular cancer therapy, TCS with cryopreservation conceived children more often (35% vs. 5%; *p* > 0.001) than those without cryopreservation. Among TCS with cryopreservation, 10% (9 out of 91) had an unfulfilled desire to have children, and 11% (10 out of 91) used their sperm for artificial insemination. The age of these patients was 34.7 ± 5.5 years (ranging from 29 to 43 years). Out of these, 30% (3 out of 10) were unsuccessful.

Among TCS without cryopreservation, 2% (3 out of 185) had an unfulfilled desire to have children. All three patients (with ages at diagnosis of 35, 42, and 44 years) had a lower level of education (middle school or lower), an income of < 4000 €/month, and already had one child before diagnosis. Interestingly, among those TCS with cryopreservation, 10% (9 out of 91) had an unfulfilled desire for parenthood.

### Quality of life

In univariate analysis, patients who did not undergo cryopreservation exhibited a lower level of concern regarding their ability to conceive children (22.8 ± 33.7 vs. 53.8 ± 37.1, *p* < 0.001), worse scores in communication (78.9 ± 26.3 vs. 87.4 ± 16.9; *p* = 0.002), sexual activity (60.1 ± 27.2 vs. 73.8 ± 22.4; *p* < 0.0001), sexual enjoyment (75.1 ± 27.3 vs. 87.1 ± 19.0; *p* < 0.001), and sexual problems (23.4 ± 29.3 vs. 13.6 ± 22.0; *p* = 0.003). Moreover, they showed worse scores concerning their overall health (4.0 ± 1.2 vs. 3.6 ± 1.1; *p* = 0.02) (refer to Table 2). In multivariate binary logistic regression analysis, only higher age (5.4 (2.6–11.3), *p* < 0.001) and a low infertility score (4.3 (2.0–9.0), *p* < 0.001) were independent predictors for not undergoing cryopreservation in the past (refer to Table 4).

**Table 4:** Multivariate analysis concerning quality of life and no use of cryopreservation

Parameter	Univariate analysis		Multivariate analysis	
	OR	*p* value	OR	*p* value
Age at time of survey (≥ 44 years)	8.9 (4.7–16.8)	** < 0.001**	5.4 (2.6–11.3)	** < 0.001**
Charlson score (≥ 3)	2.6 (1.6–4.4)	** < 0.001**	1.7 (0.9–3.3)	0.1
EORTC infertility anxiety (≤ 33.3)	6.5 (3.6–11.9)	** < 0.001**	4.3 (2.0–9.0)	** < 0.001**
EORTC sexual activity (66.6)	0.4 (0.3–0.8)	**0.002**	0.7 (0.4–1.4)	0.3
EORTC sexual problems (16.6)	1.8 (1.0–3.0)	**0.03**	1.1 (0.5–2.1)	0.8
EORTC sexual enjoyment (83.3)	0.5 (0.3–0.9)	**0.01**	0.7 (0.3–1.4)	0.3
EORTC global health (≥ 4)	2.1 (1.3–3.6)	**0.004**	1.6 (0.8–3.1)	0.2
EORTC communication (≥ 83.3)	0.6 (0.4–1.0)	0.06		
EORTC quality of life (≥ 4)	1.5 (0.9–2.5)	0.1		
PHQ-4 (≥ 2)	1.0 (0.6–1.6)	0.9		

The values marked in bold are statistically significant values

## Discussion

In this retrospective study, we assessed sperm cryopreservation utilization before implantation of the German health insurance coverage in 2021. Moreover, this study fills a gap in current data availability by providing a contemporary overview of the cohort of cryopreservation utilization in general in Germany. We were able to show that 33% (91/188) of TCS made use of cryopreservation. 11% (10/91) of TCS with cryopreservation used their sperm for artificial insemination. 2% (3/185) of TCS without cryopreservation had an unfulfilled desire to have children. In multivariate analysis, a higher age (≥ 37 years: OR 13.1 (5.5–31.2), *p* < 0.001) and a lower school degree (middle school or less: OR 3.3 (1.6–6.9); *p* = 0.001) were independent predictors for no use of cryopreservation.

The usage rate of cryopreservation varies wide in the literature (Selter et al. 2021). A recent US study analyzing the use of fertility preservation services in tumor patients revealed a rate of 11.6% in a subpopulation of 302 TCS, giving a similar figure as in our cohort with 11% of TCS using cryopreservation for artificial insemination (Selter et al. 2021). However, an older US study showed a rate of 30% in 200 TCS (Sonnenburg et al. 2015). The issue of low usage is longstanding, with several studies attributing it to a lack of information (Korte et al. 2020b; Schover et al. 2002). Nevertheless, awareness regarding fertility protection for cancer patients has changed in recent years (Gonen 2021, Bizet et al. 2012). This shift has also prompted the introduction of reimbursement for cryopreservation by health insurance companies in Germany in 2021. However, the insufficient availability of information appears not to be the sole factor contributing to the low utilization. A European multicentric randomized intervention study analyzed information strategies of adolescent cancer patients (Balcerek et al. 2020). In this study, both the control and the intervention group showed cryopreservation rates of 32–36%. In our clinic, all TC patients received information about infertility and the offer of cryopreservation. Thus, a lack of information can be excluded.

Nevertheless, our investigations reveal a noteworthy trend: individuals with a lower educational degree (middle school or less: OR 3.3 (1.6–6.9); *p* = 0.001) are less inclined to opt for cryopreservation and are an independent predictor for no use of cryopreservation. Despite its apparent simplicity, this finding holds significance, as it underscores the necessity of addressing socio-educational factors in reproductive health decision-making, particularly given the lack of recent data and academic discussion on this topic. This corresponds to studies indicating that patients with a low level of education are less likely to make fertility preservation arrangements (Grover et al. 2016; Shnorhavorian et al. 2015). It may be necessary to provide more intensive counseling to patients with lower education levels (Pacey and Eiser 2014). Addressing information disparities and promoting clear communication on sperm freezing benefits is crucial, especially for those with lower education. Encouragingly, socioeconomic factors like income, family status, health insurance, and parenthood do not emerge as independent determinants of cryopreservation utilization.

Another independent parameter for no usage of cryopreservation is a higher age (≥ 37 years). Patients who used cryopreservation were younger (30.6 ± 7.1 vs. 42.4 ± 10.9 years, *p* = 0.001). This finding aligns with previous investigations, such as those conducted by Saito et al. (Saito et al. 2005), who reported an average age of 30.1, and Meseguer et al. (Meseguer et al. 2006), who observed a mean age of 27.1 among those choosing sperm cryopreservation. One compelling explanation for this trend is the realization among younger patients that sperm freezing represents a critical opportunity for future paternity, as reproductive capacity naturally diminishes with age (Coogan et al. 1996).

Analyzing the quality of life of TCS depending on the use of cryopreservation revealed poorer parameters for TCS without cryopreservation, particularly in relation to sexual life. TCS without cryopreservation showed significantly less sexual activity (*p* < 0.001) as well as less sexual enjoyment (*p* < 0.001) and more sexual problems (*p* = 0.003) compared to TCS making use of cryopreservation (refer to Table 2). This observation may be attributed to the higher age within this group. In multivariate analysis, only low infertility anxiety (OR 4.3 (2.0–9.0), *p* < 0.001) emerged as an independent predictor for not using cryopreservation, alongside advanced age (OR 5.4 (2.6–11.3), *p* < 0.001). This underscores the significance of individual attitudes in decision-making regarding the use of cryopreservation, in addition to social parameters such as the previously mentioned educational degree. A multicentre European study also demonstrated the impact of infertility anxiety on a group of parents and patients with respect to fertility-related wishes (Korte et al. 2020a).

With respect to our data, the question arises as to how often the sperm was used and how many patients who did not make use of cryopreservation had an unfulfilled desire to have children. In our cohort, 11% (10/91) of TCS with cryopreservation used their sperm for artificial insemination. This corresponds to a recent German study, where 9.1% (46/506) of cancer patients used their cryopreserved sperm for artificial insemination (Fernandez-Gonzalez et al. 2023). Another study demonstrated an utilization rate of 6.2% in 373 TCS (Bizet et al. 2012). A large systematic review of 30 studies with 11,798 patients showed an aggregated rate for use of cryopreserved semen of 8% in cancer patients (Ferrari et al. 2016). Meseguer et al. reported a similar pattern, with approximately 15% of male patients opting to preserve their semen samples for future use, even after several years (Meseguer et al. 2006).

Interestingly, 10% (9 out of 91) of testicular cancer survivors who underwent cryopreservation had an unfulfilled desire for parenthood (refer to Table 2). This finding is important to consider as cryopreservation is not flawless, and sperm is susceptible to damage and oxidative stress during the freezing and storage process. These processes can alter functional aspects of sperm, such as increased sperm DNA fragmentation, potentially reducing fertility rates among men (Kumar et al. 2019). It underscores the caution with which cryopreservation should be approached, and that its utilization does not guarantee the fulfillment of the desire for parenthood in every case.

Looking at the data from before 2021 in conjunction with the new German regulation since 2021, it remains questionable whether it would change anything in this collective, given that only 11% of TC patients availed themselves of this service (Bundesausschuss 2022). This new legislation raises questions about the necessity for industry expenditure and equitable distribution of resources. However, this study exclusively focuses on cryopreservation utilization among testicular cancer patients, whereas other tumor entities, particularly hematologic malignancies, may exhibit significantly higher uptake rates, advocating for expanded coverage. With remark to the cut-off age of 50 years, the new legislation seems reasonable. In our collective, the age of patients with cryopreservation was around 30.6 ± 7.1 (30 (18–53)) years. Age of patients who used the sperm for artificial insemination were 34.7 ± 5.5 (32 (29–43)) years. On the other hand, although the income of patients with cryopreservation was higher, it was no independent predictor for cryopreservation in multivariate analysis (Table 3). Thus, funding of cryopreservation could possibly increase the overall willingness to do so, but may not compensate for any disadvantages.

In general, our study is subject to several limitations. The unmet desire for parenthood is multifaceted, and discerning its origins within this cohort, whether stemming from testicular cancer therapy or factors associated with the female partner, remains elusive. Furthermore, our dataset lacks insight into individuals who are presently single but might encounter this concern in the future. Moreover, there might be a more intricate decision-making process regarding cryopreservation in TC patients, influenced by familial and social factors (Gonen 2021). Despite being a retrospective single-center study, we ensured consistent information provision and offered cryopreservation to all patients uniformly. The cohort comprises 297 TC patients, providing detailed insights into the utilization of cryopreservation, its predictors and its application in artificial insemination. In the end, 33% cryopreservation rate among survivors suggests adequacy, with no signs of undersupply. With just 1% (3/279) of testicular cancer survivors expressing an unfulfilled desire for children and not utilizing cryopreservation, our standardized offering to all patients appears successful. Despite this, these 3 individuals represent only 1% (3/279) of the overall cohort, indicating a minimal percentage of potentially missed patients.

In conclusion, our retrospective study sheds light on sperm cryopreservation utilization among testicular cancer survivors prior to the implementation of German health insurance coverage in 2021, addressing a significant gap in available data. We observed that 33% of patients opted for cryopreservation, with 11% utilizing their sperm for artificial insemination. Factors such as higher age and lower educational attainment were linked to non-utilization. Despite the availability of cryopreservation, 10% of individuals still harbored unfulfilled desires for parenthood, emphasizing the importance of managing patient expectations. Addressing socio-educational disparities and providing tailored counseling are vital for ensuring equitable access to fertility preservation options. Future research is essential to assess the impact of legislative changes on cryopreservation rates, particularly among financially vulnerable individuals. This study not only informs clinical practice but also underscores the need for proactive measures to promote equitable access to fertility preservation options for all testicular cancer patients.
